# Unlocking the microbiome

**DOI:** 10.7554/eLife.92482

**Published:** 2023-10-11

**Authors:** Rosana BR Ferreira, L Caetano M Antunes

**Affiliations:** 1 Department of Molecular Biosciences, University of Kansas Lawrence United States

**Keywords:** microbiome, fruit flies, *D. melanogaster*, yeast, bacteria, *D. melanogaster*

## Abstract

Individual species of bacteria and yeast present in the food of wild fruit flies work together to provide the nutrients needed for larval growth.

**Related research article** Mure A, Sugiura Y, Maeda R, Honda K, Sakurai N, Takahashi Y, Watada M, Katoh T, Gotoh A, Gotoh Y, Taniguchi I, Nakamura K, Hayashi T, Katayama T, Uemura T, Hattori Y. 2023. Identification of core yeast species and microbe-microbe interactions impacting larval growth of *Drosophila* in the wild. *eLife* **12**:RP90148. doi: 10.7554/eLife.90148.

Living peacefully within or on the surface of every multicellular organism is a community of microbes, known as a microbiome, that benefits the health of the host. In the human gut, for instance, these microbes help develop the immune system, provide protection against pathogens, and break down food for nutrition (Horrocks et al., 2023). Although scientists have made great strides in deciphering how these health benefits are achieved, identifying which microbes are responsible remains a major challenge in the field, particularly in higher organisms such as humans.

An alternative is to study organisms that have less complex microbiomes, such as fruit flies. Previous work has shown that four bacterial families make up 90% of the bacteria in the fruit fly microbiome, and only 14 families account for the other 10% (Chandler et al., 2011). Yeast are also an important part of the microbiome. Similar to bacterial populations, the diversity of yeast is also limited, with a single genus making up 59% of the yeast species present (Chandler et al., 2012). In natural habitats, wild fruit flies feed on fruit that is broken down, or ‘fermented’, by bacteria and yeast, which are then ingested by the fruit fly, causing them to become part of the fly’s microbiome. This community of microbes has been shown to generate nutrients that are critical for larval development (Shin et al., 2011; Storelli et al., 2011; Broderick and Lemaitre, 2012). Now, in eLife, Yukako Hattori from Kyoto University and JST FOREST – including Ayumi Mure as first author – report how individual members of the microbiome associated with the fruit fly *Drosophila melanogaster* contribute to larval development (Mure et al., 2023).

To identify which microbes are present during larval development, the team (who are based at various institutes in Japan) placed freshly peeled bananas near their homes to attract wild fruit flies to lay eggs on the food. They then collected samples from the bananas two and a half days (early-stage food) and four to five days (late-stage food) into the fermentation process. Mure et al. found that the yeast and bacterial species that dominated the food changed from the early- to late-stage (Figure 1A). Notably, this transition in dietary microbes occurred even when *D. melanogaster* larvae were not present, suggesting that the shift is likely caused by other factors such as interactions among the microbes.

**Figure 1.**
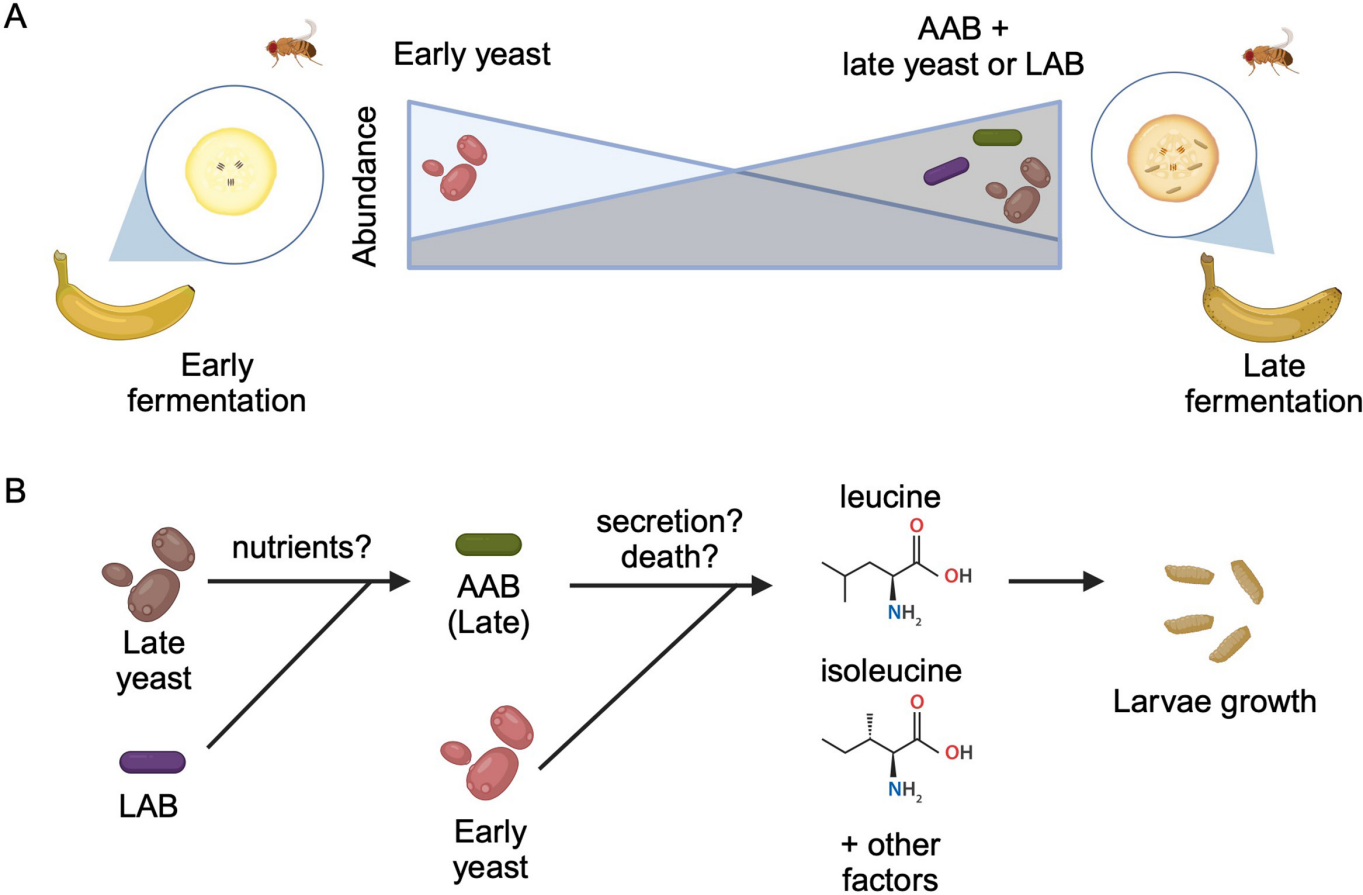
Interactions between diet, microbes, and flies. (**A**) Fruit flies consume fermenting fruits containing various bacteria and yeast, which, as a result, become part of the fly’s microbiome. Early on during fermentation (left), a specific type of yeast dominates the food (early yeast). As fermentation progresses (right), the composition shifts, and the number of early yeast cells decreases while the number of acetic acid bacteria (AAB) increases, together with other species of yeast (late yeast) and/or lactic acid bacteria (LAB). (**B**) Both acetic acid bacteria (green) and the early yeast species (pink) support larvae growth by producing two branched-chain amino acids (leucine and isoleucine) and other, unknown factors. Initially, the early yeast cells provide these amino acids. As fermentation progresses, the rising number of lactic acid bacteria (purple) and late yeast cells (brown) increases the growth of acetic acid bacteria, which take over generating the amino acids and other factors the larvae need to grow. Exactly how these nutrients are provided is still unclear, but may involve active secretion by live cells or passive release through cell death.

Image credit: Figure created using BioRender.com.

Next, Mure et al. set out to determine which of the microbes they had identified were involved in larval development. Various species of bacteria and yeast were isolated from the fermented food, and added individually or in combination to a sterile culture medium that is prepared with bananas. Larvae that were free from microbes were then introduced to the cultures to see how ingestion of the different microbial species affected the rate and timing of pupariation, which is when larvae stop crawling and surround themselves with a pupal case before undergoing metamorphosis. Larvae fed on the yeast species that dominated early fermentation (*Hanseniaspora uvarum*) exhibited high rates of pupariation, whereas the yeast species from the late-stage (*Pichia kluyveri* and *Starmerella bacillaris*) were unable to effectively promote larval growth.

Mure et al. also observed high levels of pupariation when the acetic acid bacterium *Acetobacter orientalis* from late-stage food was provided in combination with yeast or lactic acid bacteria. Further experiments suggested that *A. orientalis* needs other microbes in order to grow to a high enough level to support larval development (Figure 1B). Indeed, Mure et al. found that larvae fed a daily dose of *A. orientalis* were able to effectively pupariate even in the absence of other microbes.

To further understand how yeast support larval growth, Mure et al. investigated how species from early- and late-stage food impact pupariation when they have been killed with heat, causing their nutrients and metabolites to leak out. Surprisingly, they found that yeast from the late-stage, which had previously not supported larval growth, could now promote development when introduced into the banana-like culture medium. This suggests that all of the studied yeast strains produce nutrients and metabolites that support larval growth, but those generated by the non-supportive yeast are less accessible to the larvae.

Analyzing the metabolites present in different yeasts revealed significantly higher levels of the branched-chain amino acids isoleucine and leucine in cultures of supportive species. Although supplementing the banana-like culture with these amino acids improved larval development, the larvae could still not fully grow into adulthood. This suggests that other unidentified nutrients provided by the supportive yeasts must be playing a role.

More studies identifying which microbes are responsible for certain biological effects will be fundamental to truly understand the impact microbial communities have on host health. Furthermore, this work highlights the complexity of interactions that occur between microbes from the diet and the host microbiome. In the future, knowledge created by these studies could be used to manipulate the composition of microbiomes for specific benefits, ranging from improving human health to altering the behavior of pollinating insects.

## References

[bib1] Broderick NA, Lemaitre B (2012). Gut-associated microbes of *Drosophila melanogaster*. Gut Microbes.

[bib2] Chandler JA, Lang JM, Bhatnagar S, Eisen JA, Kopp A (2011). Bacterial communities of diverse *Drosophila* species: ecological context of a host-microbe model system. PLOS Genetics.

[bib3] Chandler JA, Eisen JA, Kopp A (2012). Yeast communities of diverse *Drosophila* species: comparison of two symbiont groups in the same hosts. Applied and Environmental Microbiology.

[bib4] Horrocks V, King OG, Yip AYG, Marques IM, McDonald JAK (2023). Role of the gut microbiota in nutrient competition and protection against intestinal pathogen colonization. Microbiology.

[bib5] Mure A, Sugiura Y, Maeda R, Honda K, Sakurai N, Takahashi Y, Watada M, Katoh T, Gotoh A, Gotoh Y, Taniguchi I, Nakamura K, Hayashi T, Katayama T, Uemura T, Hattori Y (2023). Identification of core yeast species and microbe-microbe interactions impacting larval growth of *Drosophila* in the wild. eLife.

[bib6] Shin SC, Kim SH, You H, Kim B, Kim AC, Lee KA, Yoon JH, Ryu JH, Lee WJ (2011). *Drosophila* microbiome modulates host developmental and metabolic homeostasis via insulin signaling. Science.

[bib7] Storelli G, Defaye A, Erkosar B, Hols P, Royet J, Leulier F (2011). *Lactobacillus plantarum* promotes *Drosophila* systemic growth by modulating hormonal signals through TOR-dependent nutrient sensing. Cell Metabolism.

